# Effect of acupuncture on insomnia following stroke: study protocol for a randomized controlled trial

**DOI:** 10.1186/s13063-016-1670-0

**Published:** 2016-11-16

**Authors:** Yan Cao, Xuan Yin, Francisca Soto-Aguilar, Yiping Liu, Ping Yin, Junyi Wu, Bochang Zhu, Wentao Li, Lixing Lao, Shifen Xu

**Affiliations:** 1 Shanghai Municipal Hospital of Traditional Chinese Medicine, Shanghai University of Traditional Chinese Medicine, Shanghai, 200071 China; 2 University of Maryland School of Medicine, Baltimore, MD 21201 USA; 3 School of Chinese Medicine, the University of Hong Kong, Hong Kong, China

**Keywords:** Acupuncture, Insomnia, Stroke, Randomized controlled trial

## Abstract

**Background:**

The incidence, mortality, and prevalence of stroke are high in China. Stroke is commonly associated with insomnia; both insomnia and stroke have been effectively treated with acupuncture for a long time. The aim of this proposed trial is to assess the therapeutic effect of acupuncture on insomnia following stroke.

**Methods:**

This proposed study is a single-center, single-blinded (patient-assessor-blinded), parallel-group randomized controlled trial. We will randomly assign 60 participants with insomnia following stroke into two groups in a 1:1 ratio. The intervention group will undergo traditional acupuncture that achieves the *De-qi* sensation, and the control group will receive sham acupuncture without needle insertion. The same acupoints (DU20, DU24, EX-HN3, EX-HN22, HT7, and SP6) will be used in both groups. Treatments will be given to all participants three times a week for the subsequent 4 weeks. The primary outcome will be the Pittsburgh Sleep Quality Index. The secondary outcomes will be: the Insomnia Severity Index; sleep efficacy, sleep awakenings, and total sleep time recorded via actigraphy; the National Institutes of Health Stroke Scale; the Stroke-Specific Quality of Life score; the Hospital Anxiety and Depression Scale. The use of estazolam will be permitted and regulated under certain conditions. Outcomes will be assessed at baseline, 2 weeks after treatment commencement, 4 weeks after treatment commencement, and at the 8-week follow-up.

**Discussion:**

This proposed study will contribute to expanding knowledge about acupuncture treatment for insomnia following stroke. This will be a high-quality randomized controlled trial with strict methodology and few design deficits. It will investigate the effectiveness of acupuncture as an alternative treatment for insomnia following stroke.

**Trial registration:**

Chinese Clinical Trial Registry identifier: ChiCTR-IIC-16008382. Registered on 28 April 2016.

## Background

The Global Burden of Disease Study (2013) found that cerebrovascular disease was the third leading cause of disability-adjusted life years worldwide [1]. Cerebrovascular disease is a major chronic disease in China, ranked third among the most common causes of death for urban residents and first for rural residents [2]. Stroke morbidity in China is 89.6–314 out of every 100,000 in men and 76.7–212.2 out of every 100,000 in women [3]. Stroke can be divided into two major types based on the type of pathological process: hemorrhagic stroke and ischemic stroke. The incidence of hemorrhagic stroke in China declined by 1.7% over a recent 21-year period, but that of ischemic stroke increased by 8.7% annually [4]. Common post-stroke complications include sleep disorders (SD), such as insomnia, sleep apnea, and daytime sleepiness [5], and SD continues to be the most unrecognized modifiable risk factor for stroke [6]. Untreated SD causes cognitive dysfunction, altered mood, sleepiness, and fatigue, and may impede stroke rehabilitation, lengthen hospital stay, influence stroke outcomes, and increase the possibility of stroke recurrence [7].

One of the most common SD associated with stroke is insomnia [5]. Insomnia is characterized by difficulty in falling asleep and staying asleep, early morning awakening, and nonrestorative sleep. The primary pharmacological treatments for insomnia are benzodiazepines, nonbenzodiazepine sedatives, and melatonin agonists. Although effective pharmacologic treatments for insomnia are available, these medications often cause side effects such as residual daytime sedation, drowsiness, dizziness, lightheadedness, cognitive impairment, motor incoordination, and dependence [8]. The mainstay of nonpharmacologic intervention for insomnia is cognitive behavioral therapy (CBT). However, CBT often causes an acute reduction in total sleep time (TST) during the first few weeks of treatment, which means that SD improvements with CBT require long-term implementation [9].

Faced with the limitations of currently available insomnia treatments, more people are seeking complementary and alternative medicine options. According to the theory of traditional Chinese medicine, acupuncture provides overall coordination to help the body achieve a state of relative equilibrium of yin and yang, restoring its physiological function and regulating the sleep-awake cycle. Recently, the frequency of visits to acupuncturists has markedly increased in the United States [10]; however, there has been little focus on research into acupuncture for insomnia following stroke. Two recent randomized controlled trials (RCTs) had shown that insomnia following stroke was positively affected by intradermal acupuncture and low-frequency electric stimulation at acupoints [11, 12]. However, these RCTs had methodological deficits, including problems with randomization, lack of sample size estimation, blinding issues, no mention of acupuncturist certification, and no use of the intention-to-treat (ITT) analysis [13]. These design deficits make their findings far from conclusive. More high-quality clinical RCTs are required to investigate the effectiveness of acupuncture treatment for insomnia following stroke. In this proposed RCT, we aim to investigate the effectiveness of acupuncture for insomnia following stroke.

## Methods/design

### Hypotheses

1. Participants with insomnia following stroke treated with real acupuncture will have greater improvement in sleep quality and other related symptoms than those treated with sham acupuncture
2. The sham acupuncture procedure can be successfully implemented in this RCT in which participants will be unaware of the group assignment

### Objectives

1. To compare improvement in sleep quality recorded using the Pittsburgh Sleep Quality Index (PSQI) between the intervention group and control group
2. To compare changes between the intervention group and control group in the Insomnia Severity Index (ISI), actigraphy, the National Institutes of Health Stroke Scale (NIHSS), the Stroke-Specific Quality of Life (SS-QOL), and the Hospital Anxiety and Depression Scale (HADS)
3. To evaluate the design of the sham acupuncture procedure

### Design

This is a single-center, patient-assessor-blinded, parallel-group RCT that conforms to the Consolidated Standards of Reporting Trials [14] and Standards for Reporting Interventions in Clinical Trials of Acupuncture [15] guidelines for acupuncture studies. Eligible patients will be randomly divided into the intervention group and the control group in a 1:1 allocation ratio.

**Fig. 1:**
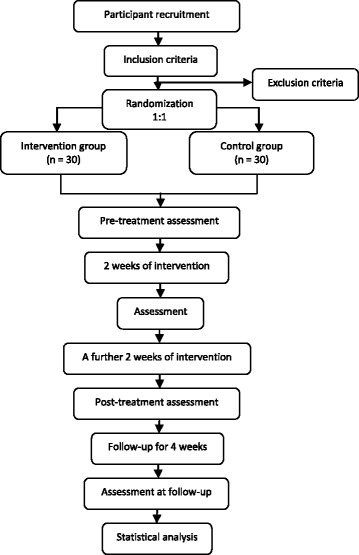
Study process

The flowchart of the study process is detailed in Fig. 1. The timing of treatment visits and data collection is detailed in Table 1.

**Table 1:** Timing of treatment visits and data collection

	Baseline	Treatment phase			Follow-up phase
		Week 0	Week 2	Week 4	Week 8
Sign informed consent		×			
Medical history	×				
Randomization		×			
Intervention		×	×	×	
Primary outcome					
PSQI	×			×	×
Secondary outcomes					
ISI	×		×	×	×
Actigraphy	×		×	×	×
NIHSS		×	×	×	×
SS-QOL		×	×	×	×
HADS		×	×	×	×
Estazolam dose			×	×	×
Adverse events			×	×	×

*HADS* Hospital Anxiety and Depression Scale, *ISI* Insomnia Severity Index, *NIHSS* National Institutes of Health Stroke Scale, *PSQI* Pittsburgh Sleep Quality Index, *SS*-*QOL* Stroke-Specific Quality of Life

### Recruitment

All participants will be recruited through advertisements on micromessage (Wechat) and hospital bulletin boards in Shanghai Municipal Hospital of Traditional Chinese Medicine in Shanghai, China. If they show interest in participating, they can call the researchers with the phone number reserved. The researcher in charge of recruitment will explain the study in detail, and outline the potential benefits of this RCT to patients with insomnia following stroke. Patients who show interest in participating will be assessed according to the inclusion and exclusion criteria on their first visit. Once they agree to participate in this RCT, they will be asked to sign written informed consent before undergoing acupuncture intervention.

### Participants

Inclusion criteria: men or women aged 18–75 years who (1) meet the diagnostic criteria of ischemic stroke according to the guidelines for the diagnosis and treatment of acute ischemic stroke in China (2014) [16] in the past 2 weeks to 2 years, (2) meet the diagnostic criteria of insomnia according to the *Diagnostic and Statistical Manual of Mental Disorders*, *Fifth Edition* [17] for at least 1 month, (3) PSQI score ≥ 6, (4) ISI score ≥ 8, (5) demonstrate no problems with communication, both visual and auditory (reading, writing, hearing, speaking, and seeing), and (6) sign a written informed consent for participation in the RCT.

Exclusion criteria: (1) those who have experienced insomnia before their stroke, (2) a history of sleep apnea syndrome, (3) a history of severe neural or psychiatric disorder or major neuropsychiatric disorder (such as autism, learning disorder, or mental retardation), (4) severe primary disease of the cardiovascular, hepatic, renal, or hematopoietic system, (5) infection or inflammation at the acupoints, (6) hemorrhagic disease or anticoagulant intake, (7) those who have used Western and/or Oriental medicine in the past 2 weeks for depression, or anxiety, (8) those who have received acupuncture for insomnia in the past month, (9) those who have participated in any other clinical trial in the past 3 months, (10) those who are pregnant or breastfeeding, and women of childbearing age who are not using proper birth control, and (11) refusal to wear a wristwatch-like actigraph while sleeping.

Dropout criteria: (1) development of serious disease (such as heart disease, pneumonia, or stroke) during the observation period, (2) receiving other treatments for insomnia (such as other Western medicine, Oriental medicine, moxibustion, or massage) during the observation period, (3) participants who present with serious adverse events, (4) participants who refuse to continue to participate in the RCT, and (5) those lost to follow-up.

### Intervention

The participants from outpatient departments will receive acupuncture treatments in the outpatient clinic of the acupuncture department three times per week for 4 weeks. The inpatients will receive acupuncture treatment three times per week at the time of hospitalization, and will then receive outpatient treatment three times per week for 2 weeks. In each treatment session, every patient will be placed in a separate quiet space and asked to wear an eye-patch. Each treatment session will be 20 min. The treatment course will last for 4 weeks, and the follow-up period will be 4 weeks. All participants will receive standard Western medicine therapy for stroke, limb function rehabilitation therapy, and clinical nursing in Shanghai Municipal Hospital of Traditional Chinese Medicine. To improve adherence to intervention protocols, all participants will receive intervention and assessments by telephone reservations, and be given some financial subsidies after follow-up.

Patients in both groups will be treated with stainless steel needles (0.25 × 25 mm and 0.30 × 40 mm; Wuxi Jiajian Medical Material Co., Ltd., Wuxi, China) at the same acupoints, including *Baihui* (DU20), *Shenting* (DU24), *Yintang* (EX-HN3), bilateral *Anmian* (EX-HN22), bilateral *Shenmen* (HT7), and bilateral *Sanyinjiao* (SP6). The acupuncturist will use 0.25 × 25-mm needles for acupoints DU20, DU24, EX-HN3 and HT7, and 0.30 × 40-mm needles for acupoints EX-HN22 and SP6 (Table 2).

**Table 2:** Details of interventions

	Intervention group	Control group
Acupoints	DU20, DU24, EX-HN3, EX-HN22, HT7, SP6	DU20, DU24, EX-HN3, EX-HN22, HT7, SP6
Depth of needle insertion	DU20, DU24, EX-HN3: 10 mm	No needle insertion
	EX-HN22, SP6: 15 mm	
	HT7: 5 mm	
Needle retention time	20 min	20 min
Needle type	Stainless steel	Blunt stainless steel
	(0.25 × 25 mm or 0.30 × 4 0 mm; Wuxi Jiajian, Wuxi, China)	(0.25 × 25 mm or 0.30 × 40 mm; Wuxi Jiajian, Wuxi, China)
Frequency and duration of treatment sessions	Three times a week for the subsequent 4 weeks	Three times a week for the subsequent 4 weeks
Needle stimulation	*De-qi* sensation	Without *De-qi* sensation

### The intervention group

The intervention group will be treated with the real tube-needling method, while lying in the supine position and wearing an eye-patch. After routine skin sterilizing, DU20, DU24, and EX-HN3 will be punctured obliquely 10 mm deep into the skin; EX-HN22 and SP6 will be punctured 15 mm deep in a direction perpendicular to the skin, and HT7 will be punctured perpendicularly to a depth of 5 mm. Licensed and experienced acupuncturists will conduct needle manipulation including lifting, thrusting, and rotating to obtain the proper needling sensation (*De-qi* sensation). Then they will stick a 2 × 2-cm piece of tape on the skin beside each needle. After a 20-min retention period, the needles and tapes will be removed simultaneously.

### The control group

The control group will receive sham acupuncture treatment. Before each session of sham acupuncture, we will prepare plastic tubes with a blunt needle inside each one. The acupuncturist will put the tube needle-end-up on the skin, and knock the tube slightly. The needles with blunt tips will not be inserted into the skin, but will create a pricking sensation similar to real acupuncture. After removing the tubes, the blunt needles will be stuck at the acupoints with 2 × 2-cm pieces of tape. After a 20-min retention period, the needles and tapes will be removed simultaneously.

### Outcomes

#### Primary outcomes

The primary outcomes of this study will be the changes in the PSQI between baseline, 4 weeks after treatment commencement, and the 8-week follow-up. All questionnaires will be in Chinese.

- Pittsburgh Sleep Quality Index
  - The PSQI is a self-rated questionnaire used to assess sleep quality and disturbances over a 1-month period. It comprises 19 self-rated items and five other-rated items [18]. The scores for the 19 self-rated items and five other-rated items are not included in the total score. Except for the first item, the remaining 18 items include the seven following subscales: subjective sleep quality, sleep latency, sleep duration, habitual sleep efficacy (SE), sleep disturbances, use of sleeping medication, and daytime dysfunction. Each subscale is rated from 0 to 3, and the accumulated scores of the seven subscales constitute the total score of the PSQI (0–21). A higher score indicates a more severe SD.

#### Secondary outcomes

- Secondary outcomes refer to the ISI score, the NIHSS score, the SS-QOL, the HADS, and the dose of estazolam during the 8-week trial at baseline, 2 weeks after treatment commencement, 4 weeks after treatment commencement, and the 8-week follow-up. All questionnaires will be presented in Chinese
- Insomnia Severity Index
  - The ISI is a Likert 5-point self-rated questionnaire designed to assess the nature, severity, and impact of insomnia. It includes seven items, with the sum score ranging from 0 to 28 [19]. A higher score suggests more severe sleep disturbance. ISI categories are: clinically significant insomnia (score 0–7), subthreshold insomnia (score 8–14), moderate clinical insomnia (score 15–21), and severe clinical insomnia (score 22–28)
- Actigraphy
  - The wActiSleep-BT actigraph (Actigraph LLC, Pensacola, FL, USA) is a noninvasive, small, wristwatch-like device that will be worn on the patient’s nondominant wrist. Patients will be instructed to wear the actigraph for two consecutive nights before each assessment time point. The patients’ motion will be recorded in 1-min epochs during sleep and stored continuously as data of SE, sleep awakenings, and TST. The analysis of sleep quality will be performed by dedicated ActiLife 6 data analysis software (ActiGraph LLC)
- National Institutes of Health Stroke Scale
  - The NIHSS is a stroke-specific quantitative scale used to assess neurological deficits in terms of consciousness, eye movements, visual fields, facial palsy, motor and sensory impairments, ataxia, language function, and neglect. A higher score indicates a more severe neurological deficit [20]. Each participant will be assessed by a trained, certified investigator at baseline, 2 weeks after treatment commencement, 4 weeks after treatment commencement, and at the 8-week follow-up
- Stroke-Specific Quality of Life
  - The SS-QOL is used to assess the health-related quality of life of stroke patients in the past week [21]. The SS-QOL consists of 49 items, with 12 domains of energy, family roles, language, mobility, mood, personality, self-care, social roles, thinking, upper extremity function, vision, and work/productivity. The total score is the sum of the 12 domain scores. A higher score indicates better function
- Hospital Anxiety and Depression Scale
  - The HADS is a validated, widely-used, 14-item self-report scale designed to briefly measure current anxiety and depressive symptoms in nonpsychiatric hospital patients [22]. It excludes somatic symptoms, therefore avoiding potential confounding factors. The HADS comprises two independent seven-item subscales for anxiety and depression
- Estazolam dose
  - Throughout this RCT, participants will be allowed to take 0.5–2 mg estazolam when they experience difficulty falling asleep for more than two consecutive days, or when they feel seriously ill because of insomnia symptoms. The dose should be recorded in the paper

### Sample size

The sample size calculation for our RCT is based on the change in the PSQI score. According to previous research [23], a sample size of 24 participants should be recruited in each group. Assuming about a 20% dropout rate, we estimate that a total of 60 participants should be recruited for this RCT.

### Randomization

We plan to use the random block method. An independent researcher, who has no contact with any participant, will use SPSS version 23.0 software (SPSS Inc., Chicago, IL, USA) to generate a random number table to divide the eligible participants in a 1:1 ratio into the intervention group and the control group. The random allocation sequence will be generated in a block of 3. The researcher will then make random allocation cards and seal each card in an opaque envelope whose number is the same as the number on the card. According to the time order in which patients are asked to go to the hospital, another researcher will arrange the patients into different groups and inform the acupuncturists of the group assignment.

### Blinding method

This is a single-blinded (patient-assessor-blinded) study. Participants will be asked to wear an eye-patch in a separate quiet space before treatment, and will remain blinded during the whole treatment. Except for the acupuncturists, all other researchers (including the statisticians, outcome assessors, and data analysts) will be blinded to the group assignment. We will attempt to validate the successful implementation of the blinding method. At first treatment, all patients will be told that the needling sensation that they are experiencing is the real sensation of acupuncture. In addition, all researchers will undergo training on the specifications of this RCT before commencement, and will strictly adhere to the separation principle of each department. The primary blinding method was successfully implemented by Lao et al. [24] in a trial to evaluate the efficacy of acupuncture treatment for postoperative oral surgery pain. We used this report as a reference to improve the design of our RCT and to ensure successful completion.

### Safety assessment

Participants will be asked to report any adverse event or related information after acupuncture treatment. The detail of every adverse event will be measured by Dr. Francisca Soto-Aguilar and reported in the Case Report Form. Adverse events include any unfavorable or unintended signs, symptoms, or diseases occurring during the whole RCT.

### Statistical analysis

All data will be analyzed according to the ITT principle to reduce deviation. The Student’s *t* test will be used to compare differences in measurement data. The rank sum test will be used to deal with ranked data, and the chi-squared test will be used to analyze categorical data. Analyses will be performed with SPSS version 23.0 software (SPSS Inc., Chicago, IL, USA).

### Monitoring

To guarantee the quality of this RCT, it will be carried out by Shanghai Municipal Hospital of Traditional Chinese Medicine. We will promptly input data from this RCT on the ResMan website at http://www.chictr.org.cn/index.aspx. As the Data Monitoring Team to identify problems in the project, examine collected data, and control bias, The Clinical Research Center of Drugs of the Shanghai University of Traditional Chinese Medicine will have access to these interim results and make the final decision to terminate the trial. A qualified clinical trial expert will be invited to monitor this RCT.

### Clinical trial registration

We applied for registration of this RCT in the Chinese Clinical Trial Registry. This will reduce all kinds of bias in clinical research, increase the transparency of trials, ensure the high quality of clinical trials and test processes, and the credibility of results.

## Discussion

Acupuncture treatment has been widely used to reduce the symptoms of insomnia [25]. There is minimal theoretical research or clinical RCTs on acupuncture for insomnia following stroke. This RCT will investigate the effectiveness of acupuncture in reducing insomnia following stroke. We are attempting to conduct a clinical RCT with adequate design and few deficits. We designed the sham acupuncture method without needle insertion to assess the specific effects of acupuncture rather than its placebo effect for treating insomnia following stroke. Furthermore, in contrast to the subjective assessments made in most trials, we will use actigraphy in our RCT to objectively assess the physiological changes in patients with insomnia. We will use the ITT principle to handle statistical data to reduce deviation of the RCT.

There are some inevitable limitations in this RCT. For example, it is hard to make the RCT double-blinded. The participants, the assessor, and the statistical expert will be blinded to the group assignment in this RCT; however, it will be impossible to blind the acupuncturist. This RCT is also restricted to a single-center hospital. Furthermore, although actigraphy is suitable for measuring nocturnal restlessness, it cannot differentiate between sleep stages or score rapid eye movement sleep, which may have helped to explain the mechanism of acupuncture for treating insomnia following stroke. In further studies, we may consider a multicenter clinical trial, using polysomnography as an assessment method.

### Trial status

We started recruiting participants in July 2016.

## Abbreviations

CBT: Cognitive behavioral therapy
HADS: Hospital Anxiety and Depression Scale
ISI: Insomnia Severity Index
ITT: Intention-to-treat
NIHSS: National Institutes of Health Stroke Scale
PSQI: Pittsburgh Sleep Quality Index
RCT: Randomized controlled trial
SD: Sleep disorders
SE: Sleep efficacy
SS-QOL: Stroke-Specific Quality of Life
TST: Total sleep time
